# Inhibition of HIV derived lentiviral production by TAR RNA binding domain of TAT protein

**DOI:** 10.1186/1742-4690-2-71

**Published:** 2005-11-17

**Authors:** Michael Y Mi, Jiying Zhang, Yukai He

**Affiliations:** 1 Departments of Dermatology and Immunology, University of Pittsburgh, School of Medicine. 190 Lothrop St, Suite 145, Pittsburgh, PA 15261, USA

## Abstract

**Background:**

A critical step in the production of new HIV virions involves the TAT protein binding to the TAR element. The TAT protein contains in close proximity its TAR RNA binding domain and protein transduction domain (PTD). The PTD domain of TAT has been identified as being instrumental in the protein's ability to cross mammalian cell and nuclear membranes. All together, this information led us to form the hypothesis that a protein containing the TAR RNA binding domain could compete with the native full length TAT protein and effectively block the TAR RNA binding site in transduced HIV infected cells.

**Results:**

We synthesized a short peptide named Tat-P, which contained the TAR RNA binding and PTD domains to examine whether the peptide has the potential of inhibiting TAT dependent HIV replication. We investigated the inhibiting effects of Tat-P *in vitro *using a HIV derived lentiviral vector model. We found that the TAT PTD domain not only efficiently transduced test cells, but also effectively inhibited the production of lentiviral particles in a TAT dependent manner. These results were also supported by data derived from the TAT activated LTR-luciferase expression model and RNA binding assays.

**Conclusion:**

Tat-P may become part of a category of anti-HIV drugs that competes with full length TAT proteins to inhibit HIV replication. In addition, this study indicates that the HIV derived lentiviral vector system is a safe and reliable screening method for anti-HIV drugs, especially for those targeting the interaction of TAT and TAR RNAs.

## Background

The HIV TAT protein is a key regulator of viral replication [1]. Binding of the TAT protein to the TAR element, a 59 nt sequence at the 5' end of nascent RNA, is the first critical step for producing full length HIV RNA. The transcription of HIV RNA from both integrated and non-integrated HIV genome is dependent on TAT protein [2]. Thus, interruption of this TAT-TAR interaction has been considered as a possible way to inhibit HIV replication [3]. TAR RNA decoys have been shown to be able to interfere with the binding of TAT proteins to native TAR elements, thus inhibiting HIV replication [4-6]. However, delivery of oligonucleotides *in vivo *is not trivial. Conversely, small synthetic substances, or short TAT peptides mimicking the TAT and TAR RNA binding domains have been shown to be promising inhibitors of HIV replication [7,8]. Furthermore, a different fragment of the TAT protein could compete for the binding site of the CXCR4 receptor on T cells and inhibit HIV entry [9]. Recently, several research groups have identified the TAR RNA binding domain of the TAT protein to be an arginine rich region (aa 49–59) [10,11]. In addition, TAT has been found to contain a protein transduction domain (PTD) that is able to cross cell membranes to freely enter cells [12]. Furthermore, this TAT PTD also has the ability to deliver big and small molecules into target cells and cell nuclei [13-15]. We have found that the TAT PTD and the TAR RNA binding domain are located in the same region of the TAT protein. The close proximity of these two properties led us to hypothesize that the sequence of this region could serve as a decoy by competing with full-length native TAT proteins. Blocking the interaction between native TAT proteins and the TAR RNA could subsequently inhibit viral replication.

The lack of access to hazardous HIV laboratories has hindered anti-HIV drug development. For this reason, it is important to explore substitute HIV models. One option is to use non-human lentiviral models, such as equine infectious anemia virus (EIAV) [16], feline immunodeficiency virus (FIV) [17], bovine immunodeficiency virus (BIV) [18], and simian immunodeficiency virus (SIV) [19,20]. While these animal models have revealed important lentivirus replication and pathogenesis mechanisms, some discrepancies still exist between animal and human lentiviruses (HIV). For instance, the above animal models may not reflect the actual HIV life cycle in humans.

A different research method is represented by the HIV derived recombinant lentiviral vector system, which was developed for human gene therapy purposes [21]. First generation HIV based lentiviral vectors were generated by deleting the viral envelope gene (env) and replacing it with the vesicular stomatitis virus glycoprotein (VSV-G) gene to eliminate viral tropism for T lymphocytes and macrophages. In addition, gag, pol, and other regulatory HIV proteins were encoded on separate plasmids that were then co-transfected into the target cells. To improve on safety in second generation viral vectors, the accessory proteins encoding the nef, vif, vpu, and vpr genes were further deleted to reduce chances of generating replication competent recombinants [22]. However, the TAT and REV proteins were still required for producing lentiviral vectors and were provided by separate plasmids. In third generation lentiviral vectors, the introduction of strong chimeric promoters drove the full length RNA without the assistance of TAT [23]. Because second generation lentiviral vectors are dependent on TAT, we should be able to design experiments to examine anti-HIV approaches that target the TAT protein. Simultaneously, the third generation lentiviral vectors that are TAT independent can be used as controls. As described above, the use of theses vectors represent a strong biosafety profile. Additionally, by coding a marker gene into the recombinant lentiviral vector model, such as green fluorescent protein (GFP), we can easily measure viral infectivity and titer through cell counts, rather than measuring viral load indirectly through p24 or other viral structural products.

In this study, we describe the synthesis of a short peptide named Tat-P, which shares the same sequence as the TAR-RNA binding domain and the TAT PTD domain, and this peptide was evaluated *in vitro *using the HIV derived recombinant lentiviral vector model to examine its potential for inhibiting TAT dependent HIV replication. The ultimate goal of these studies was to determine if Tat-P could cross cellular and nuclear membranes and effectively block native TAT proteins from binding to TAR-RNA.

## Results

### Tat-P and Con-P1 peptides efficiently transduced 293T cells

**Figure 1:**
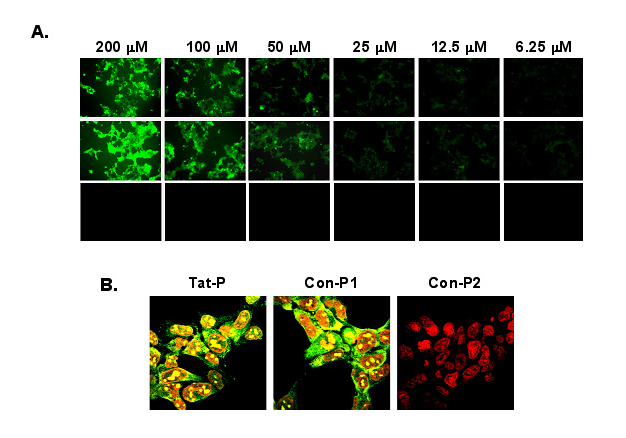
**Transduction of 293T cells by Tat-P and Con-P1**. To test the capability of Tat-P to cross 293T cell membranes, FITC labeled Tat-P, Con-P1 or Con-P2 peptides were added to 293T cells with concentrations ranged from 6.25 μM and 200 μM. Three hours later, cells were washed extensively with PBS and viewed under fluorescent microscope (Magnification ×200) (Panel **A**). In some experiments, after transduction with 200 μM peptides, 293T cells were fixed and the nuclei were counter-stained with Sytox Orange (red). Cells were then visualized under confocal microscope (Magnification ×1000) (Panel **B**).

In order to prevent native TAT proteins from binding to TAR-RNA, Tat-P must have the capability of crossing cell and nuclear membranes. To assess the transduction efficiency of Tat-P and two control peptides, Con-P1 and Con-P2, we synthesized FITC conjugated peptides. Con-P1 was utilized as a positive control because previous studies have demonstrated that this peptide shares similar structure and cell entry properties to Tat-P, conversely, Con-P2 represented a negative control because it lacks the PTD domain and its associative cell entry capabilities [24]. The 293T cells were treated with FITC labeled peptides ranged from 6.25 μM to 200 μM for 3 hours at 37°C, and internalization of these peptides was evaluated by fluorescent microscopy. As shown in Fig. 1A, the 293T cells displayed high levels of transduction by both Tat-P and Con-P1, and that the degree of transduction for these peptides was observed to be dose dependent. Furthermore, the peptide was found in the nucleus of transduced cells when examined with confocal microscopy (Fig. 1B), suggesting that the peptide was indeed inside the cells not simply attached to the cell surface. As expected, the Con-P2 negative control peptide was unable to transduce the 293T cells. These data confirm previous reports that Tat-P can cross cell membranes to enter the cytoplasm and then the nucleus.

### Tat-P inhibited the viral production of second-generation recombinant lentiviral vector

To evaluate the blocking of HIV TAT and TAR RNA interaction as a feasible target for anti-HIV drug development and to test whether the Tat-P blocks lentiviral vector particles production, 293T cells were transfected with three plasmids providing necessary genes to package replication-defective pseudo-typed HIV particles. Twenty-four hours after the transfection, Tat-P or the control peptides Con-P1 and Con-P2, were added to the cells. If Tat-P is able to compete with full length TAT protein for binding to TAR-RNA, it should block TAT transactivation activity and thus inhibit the viral RNA transcription and lentiviral production. We utilized the following two indicators to evaluate the inhibition of recombinant lentiviral production.

#### (1) Visualization of HIV production by electronic microscopy (EM)

**Figure 2:**
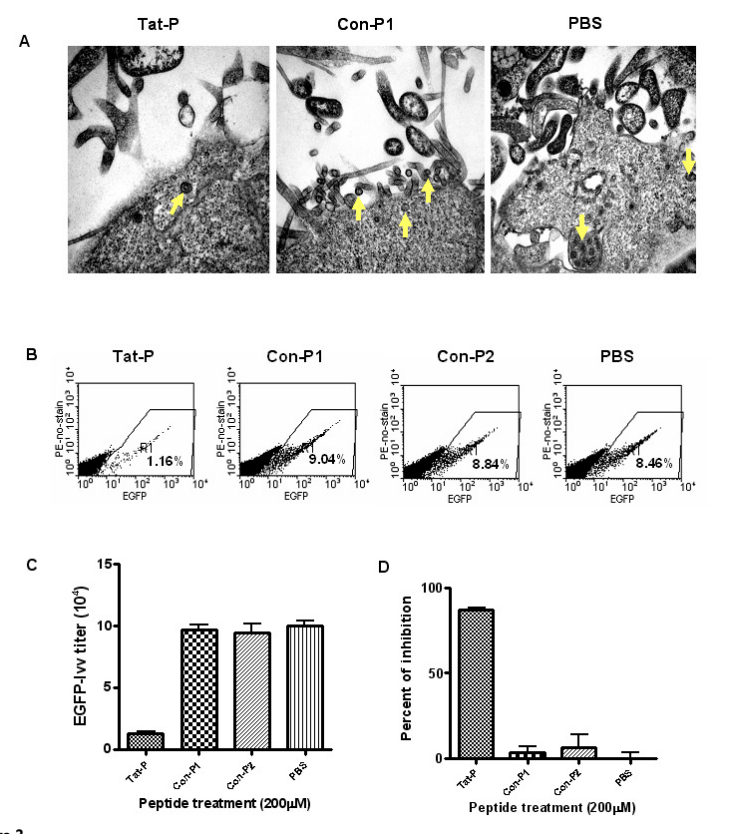
**Inhibition of recombinant lentiviral vector generation by Tat-P peptide**. Panel **A. **To visualize the genetically generated pseudo HIV particles, 293T cells were co-transfected for 24 hours with 3 plasmids: pCMV Δ8.91, pMD VSV-G, and pHR'GFP. Then 200 μM of Tat-P and Con-P1 peptides, and the same amount of PBS, were added to the 293T cells for 12 hours. The cells were then fixed and sectioned for EM imaging (Magnification × 60,000). Arrows indicate the virus particles. Panel **B **and Panel **C**. To examine the Tat-P inhibition of HIV derived recombinant lentiviral vectors, 293T cells were cotransfected with 3 plasmids for 24 hours. The medium was replaced with DMEM containing 200 μM of Tat-P, Con-P1, Con-P2 peptides or PBS. Six hours later, the supernatants containing the viral particles were collected and the vector titers were determined. A representative from three individual experiments is presented. Panel **D**. Percent inhibition was calculated using the formula (1-titer in the presence of peptide/titer in the presence of PBS) × 100.

Twenty-four hours following transfection, the media was replaced with fresh media containing 200 μM of Tat-P, Con-P1, or the same amount of PBS for 12 hours. The cells were then fixed and sectioned for transmission EM imaging. Fig. 2A shows that HIV particles were formed by the Tat-P and Con-P1 transfected 293T cells. From these EM images, the recombinant lentiviral vectors were visualized as 80~100 nm enveloped viral particles. It is important to note that the Tat-P treated cells showed formation of fewer viral particles than those of Con-P1 and the PBS treated controls.

#### (2). Reduction in lentiviral titers following addition of the Tat-P

To accurately assess the Tat-P inhibition capability, we measured the lentiviral vector titer in the cell culture supernatant generated from co-transfection in the presence or absence of peptides. As shown in Fig. 2B, cell culture supernatant from co-transfection in the presence of Tat-P generated significantly fewer number of GFP positive cells, indicating much lower lentiviral vector titer in the preparation. The vector titer was calculated based on the initial number of 293T cells when lentiviral vector was added. The lentiviral vector titer was dramatically reduced in the presence of Tat-P (Fig. 2C). The inhibition effects of Tat-P, Con-P1, and Con-P2 on the lentiviral production were calculated to be 89.8%, 4%, and 5.9% compared to PBS control, respectively (Fig. 2D), suggesting that Tat-P strongly inhibit the lentiviral production in the setting of three plasmids co-transfection approach.

### Tat-P inhibition of virus production was constant over time and the degree of inhibition was dose dependent

**Figure 3:**
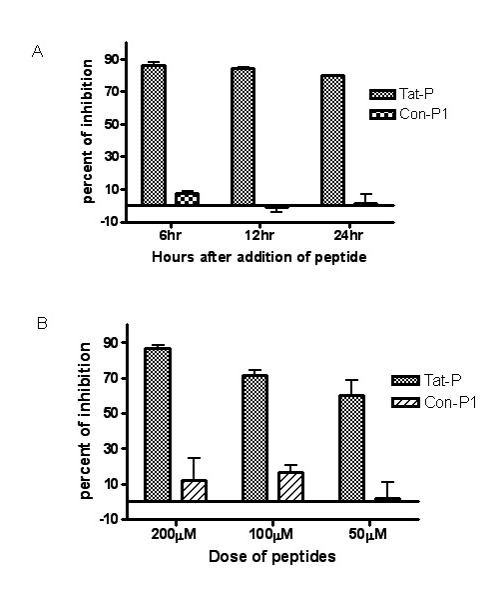
**Time and dose effects of Tat-P on recombinant lentiviral vector production**. Panel **A**. To investigate the Tat-P driven inhibition of lentiviral vector generation over time, supernatants from the viral particle producing 293T cell cultures, in the presence of 200 μM of Tat-P, Con-P1, Con-P2, and PBS, were collected at 6 hour, 12 hour, and 24 hour time points. The supernatants containing the viral particles were added to freshly cultured 293T cell and these supernatants were evaluated for virus titers. Panel **B**. To assess dose-dependency of the Tat-P inhibition activity, supernatants from the viral particle producing 293T cell cultures, in the presence of 200 μM, 100 μM, and 50 μM of Tat-P and Con-P1, were collected at the 6-hour time point. Viral titers were determined and the inhibition effects were calculated.

Tat-P inhibition effect was also demonstrated in the time point of 12 hours and 24 hours after addition of the peptide. As shown in Fig. 3A, the inhibition rates were 83% and 79%, respectively. The inhibition effect was decreased with incremental time length possibly due to the peptide degradation. In contrast, Con-P1 peptide had no inhibition effect at all time points, further suggesting the inhibition by Tat-P was specific. To evaluate the dose-response effect of the Tat-P on viral production, three different doses of Tat-P (200 μM, 100 μM and 50 μM) were utilized in the experiment. At each dose of the treatment, Tat-P inhibited the viral production quantified by flow cytometry (Fig. 3B) when compared to Con-P1 treatment and PBS control (data not shown). Compared to Con-P1 peptide, the inhibition rates of Tat-P were calculated to be 87.1% at 200 μM, 72.7% at 100 μM, and 59.2% at 50 μM.

### Tat-P did not inhibit third generation virus production

**Figure 4:**
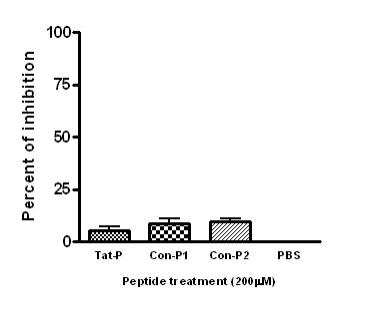
**No inhibition of third generation lentiviral vectors by Tat-P was observed**. To test that the Tat-P inhibition activity is specifically targeting HIV TAT protein, 293T cells were cotransfected with four plasmids of a third generation lentiviral vector system that is independent of the TAT protein. Then, 200 μM of Tat-P, Con-P1, and Con-P2 peptides, or a PBS control were added to the 293T cells for 6 hours. The supernatants containing the viral particles were collected and added to freshly cultured 293T cells to measure viral titers.

In this experiment, we evaluated whether the inhibition of HIV replication by Tat-P was TAT protein dependent. Since the TAT protein is not required to produce third generation recombinant lentiviral vectors, then Tat-P should not inhibit third generation viral production. 293T cells were co-transfected within a third generation (TAT independent) lentiviral vector system. After exposure to Tat-P, Con-P1, Con-P2 and PBS, the cell supernatants were measured to determine virus titers (Fig. 4). All three peptides showed low levels (<10%) of virus replication inhibition. These data strongly support that the Tat-P inhibition of virus replication present above was occurring through direct interference with the native TAT proteins and their target TAR-RNA.

### Tat-P toxicity of 293T cells did not occur at concentrations less than 400 μM

**Figure 5:**
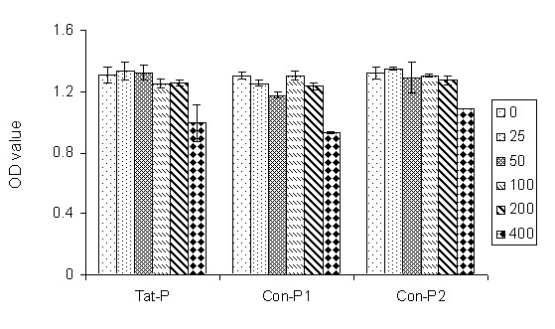
**Cytotoxicity of Tat-P on 293T cells**. To test for peptide toxicity, Tat-P, Con-P1, and Con-P2 peptides in concentrations ranging from 0 μM to 400 μM were added to 293T cells at 37°C for 6 hours, and the cell viabilities were monitored by MTT assay.

To evaluate the cell toxicity of Tat-P, escalating doses of the peptides were applied to 293 T cells. The cell viability was measured using MTT assay. Fig. 5 showed that the addition of Tat-P to the cell culture medium did not affect 293T cell viability up to 200 μM. A low level of toxicity was observed when the peptide concentration reached 400 μM. However, this toxicity level was similar to that induced by control peptides Con-P1 and Con-P2, suggesting that the inhibition of recombinant lentiviral production Tat-P is not due to the effect of cell toxicity.

### Tat-P Inhibits TAT Activated LTR-Luciferase Activity

**Figure 6:**
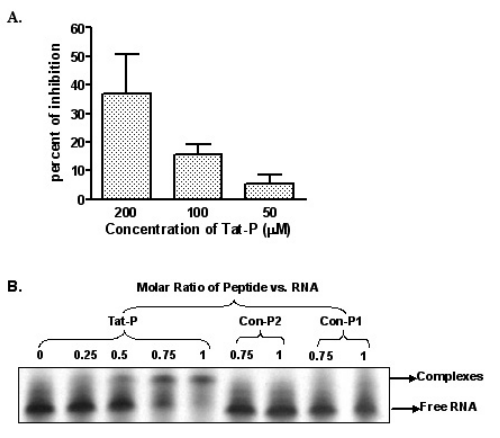
**Inhibitory effect of Tat-P on TAT activated LTR-Luciferase activity and specific TAR RNA binding by Tat-P**. Panel **A**. The TAT activated LTR-luciferase assay: 239T cells were co-transfected with the pLTR-luc and pCMV-TAT plasmids, and Tat-P and Con-P1 peptides (200 μM, 100 μM, 50 μM) were added to the cells 6 hours after transfection. The conditioned media were exchanged with fresh media containing the same amounts of peptides after 12 hours. The cells were harvested 6 hours later and processed by luciferase assay. The inhibition rates were expressed as mean ± SE. Panel **B**. The RNA binding assay: 0.25 nmol of TAR RNA was incubated with Tat-P or control peptides (Con-P1 and Con-P2) at indicated Peptide: TAR RNA molar ratio in a total 10 ul of reaction mixture for 15 minutes on ice. Free RNAs and peptide-RNA complexes were resolved by electrophoresis at 25°C on 15% polyacrylamide gels, and imaged using a fluorescent-based EMSA kit.

To verify the results that Tat-P competitively inhibited HIV based lentiviral production via interference with TAR RNA binding, HIV LTR-luciferase expression model was established by cotransfection of 293T cells with pLTR-luc and pCMV-TAT plasmids. The expression of luciferase is augmented in the presence of full length TAT protein through the binding of TAR RNA. The binding of Tat-P to TAR RNA should competitively block the interaction of TAT protein with TAR RNA, resulting in reduction of reporter gene expression. As demonstrated in Fig. 6A, luciferase activity decreased in the presence of Tat-P in a dose dependent manner. In contrast, Con-P1 peptide has no effect of luciferase gene expression, suggesting that the inhibitory effect of Tat-P ensues from competition with TAT protein and does not represent nonspecific transcription inhibitory effect.

### Tat-P Specifically Binds To TAR-RNA

We next investigated whether Tat-P's antiviral activity was due to specific binding to TAR-RNA by performing Tat-P and TAR-RNA binding assays *in vitro*. Tat-TAR-RNA complexes were formed by mixing serial dilutions of Tat-P peptide with TAR-RNA, and resolving the peptide-RNA complexes by electrophoresis on polyacrylamide gels. Fig. 6B shows that Tat-P peptides did bind to the TAR-RNA and these complexes are represented by upward shifts in the gel. As the concentration of Tat-P increased (left to right), the RNA bands showed a continuous step-up pattern indicating increasing density. No such phenomenon was observed for the control peptides, suggesting that the Tat-P peptides were binding specifically to the TAR-RNA.

## Discussion

Currently, treatments for HIV infection rely heavily on anti-viral therapies. Most of these therapies target the HIV reverse transcriptase and protease enzymes by using nucleoside analogues as enzymes inhibitors, and their combination, known as highly active antiretroviral therapy (HAART), has markedly decreased mortality and morbidity in the developed world. The disadvantages of HAART include its inability to completely eradicate HIV from the body, long-term toxicity, and eventually the emergence of drug-resistant HIV strains [25]. Furthermore, the majority of HIV carriers have limited access to anti-retrovirals (ARVs) because of high costs and problems with patient compliance. It is, therefore, vital to find new strategies for identifying anti-HIV remedies, such as new targets of viral replication, new sources of drugs, and safe anti-HIV drug screening models.

Interruption of the formation of TAT-TAR-RNA complex represents such an endeavor. Small molecules mimicking either the native TAT peptides or TAR-RNA decoys have been investigated as new approaches for inhibiting HIV replication [4-9]. The lack of access to hazardous HIV laboratories is one of major hurdles for developing anti-HIV drugs. One option to overcome this restriction is to develop lower-risk assays for use in BSL-2 laboratories. Recombinant lentiviral vectors, widely used for gene therapy research could offer a potential substitute model for evaluating the efficacy of anti-HIV drugs. This may especially be true for candidate drugs targeting the interaction between TAT and TAR-RNA, the interaction of which is required for producing second generation recombinant lentiviral vectors. Based on the observation that the short Tat-P peptide can freely enter cells and specifically bind to TAR-RNA, we investigated the hypothesis that HIV replication could be inhibited by Tat-P peptides blocking native TAT proteins from binding to the TAR-RNA, and that these studies could be performed using HIV derived lentiviral model.

In these studies, we found that Tat-P was able to transduce 293T cell membranes without significant toxicity, and that the peptides inhibited recombinant lentiviral production in a TAT dependent manner. The inhibition of recombinant lentiviral production by Tat-P likely resulted from the competitive binding with TAR-RNA and the blocking of full length TAT by Tat-P. As demonstrated in Fig. 6B, Tat-P could bind to TAR RNA. More importantly, luciferase gene expression from TAT responsive LTR promoter was inhibited by the presence of Tat-P (Fig. 6A), further suggesting that the inhibition effect of Tat-P is mediated by interference with TAT-TAR RNA interaction. Compared to the dramatic inhibition of infective lentiviral particles (Fig. 3), the inhibition of luciferase gene expression from TAT responsive promoter by Tat-P seems less dramatic (Fig. 6A). Such discrepancy was also observed previously by others using TAT responsive promoter driven CAT assay (7). One possible explanation for the difference is that there is a higher amount of TAT protein may be produced from co-transfected plasmid pCMV-TAT. Thus, the same amount of Tat-P result in less effective competitive inhibition. In contrast, TAT protein level in the generation of viral particles by co-trasnfection method may be lower since it is generated by polycistronic mRNA from plasmid pCMV Δ8.91. Alternatively, viral particle production is a multiple steps process dependent on TAT. The competitive inhibition of TAT function by Tat-P may be amplified in the subsequent steps, resulting in more dramatic reduction of infective viral particles. In addition, it is possible that production of longer RNA is more dependent on the action of TAT, whereas the shorter luciferase gene expression from LTR promoter may be less dependent on TAT. Therefore, competitive blocking of TAT interaction with TAR RNA by Tat-P results in less dramatic inhibition of luciferase activity.

The recombinant lentiviral vector model has two advantages over natural HIV cell culture model. First, it is safer and able to be conducted in most laboratories. Second, it is an alternative approach for evaluating the infective recombinant viral particles. However, it is not clear if this recombinant lentiviral vector system can also be used to screen other anti-HIV drugs, such as those that target reverse transcriptase and proteinase. The split of one HIV genome into three different plasmids in generating a lentiviral vector may create an artificial setting for studying viral pathogenesis, which may affect the anti-HIV mechanisms. Thus, the results obtained through this recombinant lentiviral vector system need to be validated by conventional *in vitro *cell culture screening methods. Nevertheless, our research has shown that the recombinant lentiviral vector *in vitro *generation model may provide an easy and safer assay for primary screenings of ARV drugs before moving on to more involved methods requiring restricted P3 facilities.

## Conclusion

Based on the above results, we draw the following conclusions: Tat-P inhibits HIV derived lentiviral production by blocking native TAT proteins from binding to TAR-RNA; genetically generated HIV models can be applied to screen anti-HIV drugs before using the high risk wild type HIV models; the results obtained from a recombinant lentiviral vector *in vitro *model need to be validated using wild type HIV cell culture methods and animal models.

## Methods

### Peptides and RNA

Tat-P (_47_YGRKKRRQRRR_57_) [10,12], Con-P1 (RRQRRTSKLMKR) [24] that shares similar structure and transduction efficiency as Tat-P, and Con-P2 (ARPLEHGSDKAT) [24] that lacks the capability of cell transduction, were synthesized (Peptide Synthesis Facility, University of Pittsburgh) using standard fmoc chemistry, then cleaved and deprotected by stirring in a 95% TFA, 2.5% triisopropylsilane, 2.5% H_2_O solution. The peptides were purified by reverse phase high performance liquid chromatography to >90% purity. Lyophilized peptides were reconstituted in PBS before use. To generate FITC labeled peptides, the fluorescein moiety (Fl) was attached via an aminohexanoic acid spacer by treating a resin-bound peptide (1.0 mmol) with FITC (1.0 mmol) and diisopropyl ethyl amine (5 mmol) in dimethylformamide (DMF; 10 ml) for 12 h [26]. Cleavage from the resin was achieved by using 95:5 trifluoroacetic acid (TFA)/triisopropylsilane. Removal of the solvent *in vacuo *gave a crude oil that was triturated with cold ether. The crude mixture thus obtained was centrifuged, the ether was removed by decantation, and the resulting orange solid was purified by RP-HPLC (H _2_O/CH_3_CN in 0.1% TFA). The TAR RNA 29mer 5'-GCCAGAUCUGAGCCUGGGAGCUCUCUGGC-3' [10] was purchased from Dharmacon (Lafayette, CO) and the RNA was purified with PAGE gel and desalted by the manufacturer.

### Transduction of 293T cells by peptides

FITC labeled Tat-P, Con-P1, and Con-P2 peptides were added to 293T cells at concentrations ranged from 6.25 μM to 200 μM and incubated at 37°C for 3 hours. The cells were washed extensively with PBS (pH.7.2) to remove excess peptides. Transduction of cells was visualized under a fluorescent microscope. To determine if the peptides were actually inside the cells, we conducted confocal microscopy study by co-staining the transduced cells with nucleus staining. 293T cells were transduced with 200 μM peptides. Three hours later, the treated cells were washed with tris buffered saline (TBS, pH 7.4) and fixed with 2% of paraformaldehyde containing 0.1% of Triton X-100 (Sigma, St. Louis, MO). The nuclei were stained with 1:2000 of Sytox Orange (Molecular Probes, Eugene, OR) and the peptide intracellular uptake was examined by confocal microscopy.

### *In vitro *generation of lentiviral vectors

The production of second and third generation recombinant lentiviral vectors was performed as described previously using a three- or four- plasmids cotransfection procedure [22,27]. For generating third-generation lentiviral vectors, 80% confluent 293T cells were transfected with plasmid DNA pLenti-EGFP-TRIP together with packaging plasmids, pLP1, pLP2, and pVSV-G, (Invitrogen, San Diego, CA) using the calcium phosphate precipitation method according to manufacturer's description (Stratagene, San Diego, CA). To produce second-generation VSV pseudo-typed lentiviral vectors, plasmid pCMV Δ8.91 expressing the core proteins and enzymes of HIV, plasmid pMD VSV-G providing the envelope protein of VSV-G, and plasmid pHR'GFP expressing the green fluorescence protein (GFP) were utilized to transfect 293T cells using the same method as above. Handling of viral vectors was according to the guideline of BSL-2+ laboratories established by the Recombinant DNA Committee of University of Pittsburgh.

### Assays for Tat-P inhibition of HIV lentiviral production

Twenty-four hours after the three plasmid transfection, media were replaced with fresh media containing different concentrations of the peptides. Cell supernatants containing viral particles were collected at 6 hour, 12 hour, and 24 hour time points to determine the viral titers by transducing 293T cells. Media collected at different time points were diluted two fold with fresh media containing 8 μg/ml of polybrene and then added to 293T cells. Two days later, cells were collected and the transduced EGFP+ cells were analyzed using flow cytometry (BD Bioscience, CA). Percentage of transduction was calculated. The quantitative data collected were expressed as mean ± SD, and the viral inhibition rates were calculated by the formula: Inhibition rate = (1 - Number of Tat-P Treated Green Cells/Number of Green Cells of a Control) × 100%.

### Visualization of viral particles using electronic microscope

Twenty-four hours after transfection, Tat-P, Con-P1, or PBS was added to the 293T cells for 12 hours. The cells were washed with PBS twice and fixed using 2% glutaraldehyde. Viral particles were examined by electronic microscope (EM) imaging.

### MTT assay for cell viability

The 293T cells were treated with medium containing peptide concentrations ranging from 0 μM to 400 μM for 6 hours at 37°C. MTT (Sigma Chemical Co, St. Louis, MO) was added to the wells at a concentration of 50 μg/ml at 37°C for 3 hours. Subsequently, the medium was aspirated, and the insoluble formazan crystals were dissolved in a solution of 10% SDS. Absorbance readings were taken at λ = 570 nm with background subtracted at λ = 630 nm [28].

### TAT dependent LTR-luciferase assay

To investigate if TAT dependent LTR-luciferase expression can be inhibited by co-delivering Tat-P, 293T cells were cotransfected with HIV LTR driven luciferase cDNA plasmid (pLTR-luc) and CMV driven full length TAT cDNA plasmid (pCMV-TAT) using a calcium phosphate precipitation method. Both plasmids are kindly provided by Dr. P. Gupta of the University of Pittsburgh, School of Public Health. At 6 hours following transfection, Tat-P and Con-P1 peptides (200 μM, 100 μM, 50 μM) were added to the cotransfected 293T cells, and the conditioned media were exchanged with fresh media containing same amounts of peptides after 12 hours. The cells were harvested 6 hours later and processed by luciferase assay (Promega, Madison WI) and the level of luciferase activity was determined at 24 hours using an illuminometer (AutoLumat LB 953, EG&G berthold). The data collected were expressed as mean ± SE, and the luciferase inhibition rate was calculated by a formula: Inhibition rate = (1 - Luminescent Units of Tat-P Treated/Luminescent Units of a Control) × 100%.

### RNA-binding assay

RNA Binding assays were performed according to a previous report [29]. Briefly, peptides and RNA were incubated together for 15 minutes on ice in 10 μl of a binding reaction mixture containing 10 mM hepes/KOH (pH 7.5), 100 mM KCl, 1 mM MgCl2, 0.5 mM EDTA, 1 mM dithiothreitol. To determine relative binding affinities, 0.25 nmol of TAR-RNA were titrated with serial dilutions of Tat-P, Con-P1 and Con-P2 (Peptide/RNA molar ratios are 0, 0.25, 0.5, 0.75, and 1). Free RNAs and peptide-RNA complexes were resolved by electrophoresis at 25°C in 15% polyacrylamide gels with 1xTBE (90 mM Tris/45 mM boric acid/1 mM EDTA) and imaged by fluorescent based Electrophoretic Mobility Shift Assay (EMSA) kit (Molecular Probes, Eugene, OR).

## List of abbreviations

HIV: Human immunodeficiency virus

TAR: Trans-activating response region

TAT: Transactivating regulatory protein

PTD: Protein transduction domain

RNA: Ribonucleic acid

Tat-P: TAT peptide

293T: A human kidney epithelial cell line

Con-P1: Control peptide one

Con-P2: Control peptide two

EM: Electron microscopy

PBS: Phosphate buffered saline

TBS: Tris buffered saline

GFP: Green fluorescent protein

MTT: 3-(4,5-dimethylthiazol-2-yl)-2,5-diphenyltetrazolium bromide

HAART: Highly active antiretroviral therapy

ARV: Anti-retroviral

FITC: Fluorescein isothiocyanate

VSV-G: Vesicular stomatitis virus glycoprotein

CMV: Cytomegalovirus

EMSA: Electrophoretic mobility shift assay EMSA

## Competing interests

The author(s) declare that they have no competing interests.

## Authors' contributions

MM designed and performed most of the experiments and wrote the manuscript. JZ provided crucial technical help for the experiments. YH supervised experimental design, experiment processes, data interpretation and writing of the manuscript.

## References

[B1] Jeang KT, Xiao H, Rich EA (1999). Multifaceted activities of the HIV-1 transactivator of transcription, Tat. J Biol Chem.

[B2] Wu Y (2004). HIV-1 gene expression: lessons from provirus and non-integrated DNA. Retrovirology.

[B3] Bannwarth S, Gatignol A (2005). HIV-1 TAR RNA: the target of molecular interactions between the virus and its host. Curr HIV Res.

[B4] Michienzi A, Li S, Zaia JA, Rossi JJ (2002). A nucleolar TAR decoy inhibitor of HIV-1 replication. Proc Natl Acad Sci USA.

[B5] Garbesi A, Hamy F, Maffini M, Albrecht G, Klimkait T (1998). TAR-RNA binding by HIV-1 Tat protein is selectively inhibited by its L-enantiomer. Nucleic Acids Res.

[B6] Banerjea A, Li MJ, Remling L, Rossi J, Akkina R (2004). Lentiviral transduction of Tar Decoy and CCR5 ribozyme into CD34+ progenitor cells and derivation of HIV-1 resistant T cells and macrophages. AIDS Res Ther.

[B7] Choudhury I, Wang J, Rabson AB, Stein S, Pooyan S, Leibowitz MJ (1998). Inhibition of HIV-1 replication by a Tat RNA-binding domain peptide analog. J Acquir Immune Defic Syndr Hum Retrovirol.

[B8] Hamy F, Felder ER, Heizmann G, Lazdins J, Aboul-ela F, Varani G, Karn J, Klimkait T (1997). An inhibitor of the Tat/TAR RNA interaction that effectively suppresses HIV-1 replication. Proc Natl Acad Sci USA.

[B9] Lohr M, Kibler KV, Zachary I, Jeang KT, Selwood DL (2003). Small HIV-1-Tat peptides inhibit HIV replication in cultured T-cells. Biochem Biophys Res Commun.

[B10] Zhao H, Li J, Jiang L (2004). Inhibition of HIV-1 TAR RNA-Tat peptide complexation using poly(acrylic acid). Biochem Biophys Res Commun.

[B11] Ruben S, Perkins A, Purcell R, Joung K, Sia R, Burghoff R, Haseltine WA, Rosen CA (1989). Structural and functional characterization of human immunodeficiency virus tat protein. J Virol.

[B12] Schwarze SR, Ho A, Vocero-Akbani A, Dowdy SF (1999). In vivo protein transduction: delivery of a biologically active protein into the mouse. Science.

[B13] Ho A, Schwarze SR, Mermelstein SJ, Waksman G, Dowdy SF (2001). Synthetic protein transduction domains: enhanced transduction potential in vitro and in vivo. Cancer Res.

[B14] Schwarze SR, Hruska KA, Dowdy SF (2000). Protein transduction: unrestricted delivery into all cells?. Trends Cell Biol.

[B15] Schwarze SR, Dowdy SF (2000). In vivo protein transduction: intracellular delivery of biologically active proteins, compounds and DNA. Trends Pharmacol Sci.

[B16] Chen C, Weisz OA, Stolz DB, Watkins SC, Montelaro RC (2004). Differential effects of actin cytoskeleton dynamics on equine infectious anemia virus particle production. J Virol.

[B17] Burkhard MJ, Dean GA (2003). Transmission and immunopathogenesis of FIV in cats as a model for HIV. Curr HIV Res.

[B18] Tok JB, Bi L, Huang S (2004). A comparative binding study of modified bovine immunodeficiency virus TAR RNA against its TAT peptide. Bioorg Med Chem Lett.

[B19] Zink MC, Clements JE (2002). A novel simian immunodeficiency virus model that provides insight into mechanisms of human immunodeficiency virus central nervous system disease. J Neurovirol.

[B20] Kumar A, Narayan O (2001). Immunization for long-term protection against AIDS using the macaque model. Virology.

[B21] Naldini L, Blomer U, Gallay P, Ory D, Mulligan R, Gage FH, Verma IM, Trono D (1996). In vivo gene delivery and stable transduction of nondividing cells by a lentiviral vector. Science.

[B22] Zufferey R, Nagy D, Mandel RJ, Naldini L, Trono D (1997). Multiply attenuated lentiviral vector achieves efficient gene delivery in vivo. Nat Biotechnol.

[B23] Dull T, Zufferey R, Kelly M, Mandel RJ, Nguyen M, Trono D, Naldini L (1998). A third-generation lentivirus vector with a conditional packaging system. J Virol.

[B24] Mi Z, Mai J, Lu X, Robbins PD (2000). Characterization of a class of cationic peptides able to facilitate efficient protein transduction in vitro and in vivo. Mol Ther.

[B25] Pereira CF, Paridaen JT (2004). Anti-HIV drug development – an overview. Curr Pharm Des.

[B26] Wender PA, Mitchell DJ, Pattabiraman K, Pelkey ET, Steinman L, Rothbard JB (2000). The design, synthesis, and evaluation of molecules that enable or enhance cellular uptake: peptoid molecular transporters. Proc Natl Acad Sci USA.

[B27] He Y, Zhang J, Mi Z, Robbins P, Falo LD (2005). Immunization with lentiviral vector-transduced dendritic cells induces strong and long-lasting T cell responses and therapeutic immunity. J Immunol.

[B28] Mai JC, Mi Z, Kim SH, Ng B, Robbins PD (2001). A proapoptotic peptide for the treatment of solid tumors. Cancer Res.

[B29] Tan R, Frankel AD (1995). Structural variety of arginine-rich RNA-binding peptides. Proc Natl Acad Sci USA.

